# Supplementary material for: Splice-Junction-Based Mapping of Alternative Isoforms in the Human Proteome
Source: Cell Rep. Author manuscript; Available in PMC 2020 Jan 15. (PMC6961840; doi:10.1016/j.celrep.2019.11.026)
Supplement: 2 [file NIHMS1546469-supplement-2.zip › DF1/PXD006675/PulmonaryVein/PulmonaryVein_22_TRIM28_RSGAAHSAMCSLTAAAASAASGSPGPGEGSAGGEK.pdf]

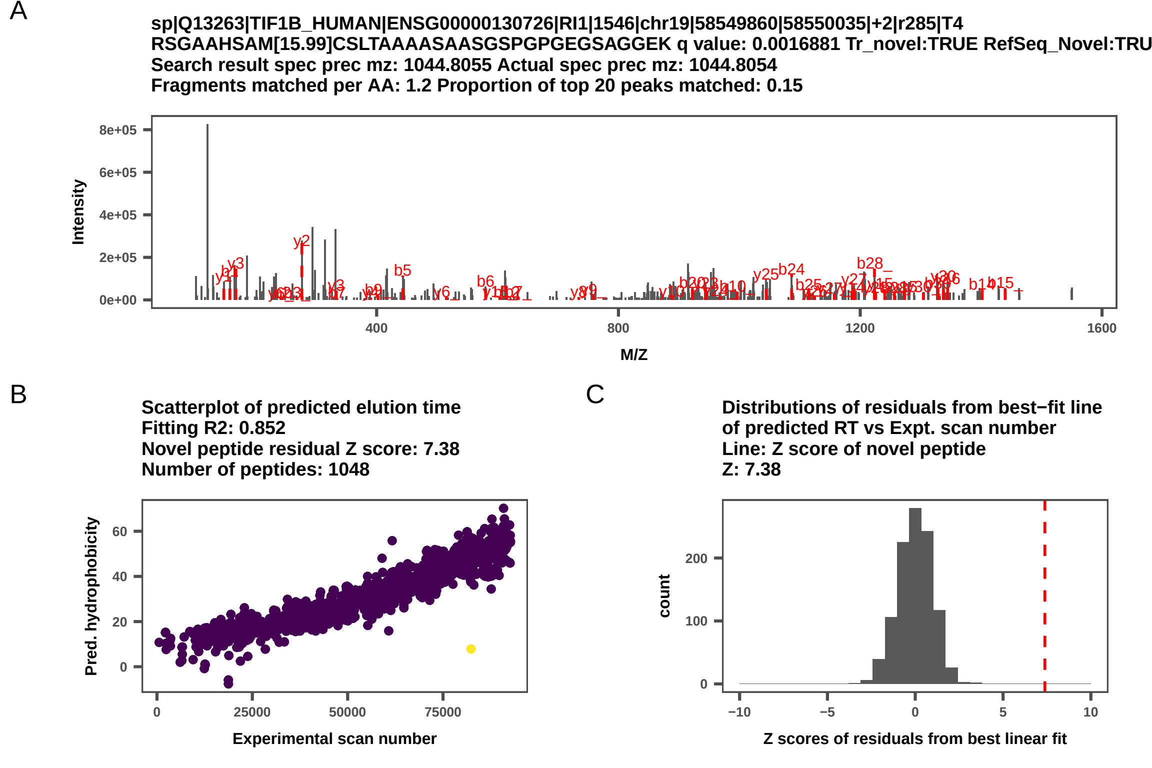
A
sp|Q13263|TIF1B_HUMAN|ENSG00000130726|RI1|1546|chr19|58549860|58550035|+2|r285|T4
RSGAAHSAM[15.99]CSLTAAAASAASGSPGPGEGSAGGEK q value: 0.0016881 Tr_novel:TRUE RefSeq_Novel:TRU
Search result spec prec mz: 1044.8055 Actual spec prec mz: 1044.8054
Fragments matched per AA: 1.2 Proportion of top 20 peaks matched: 0.15
Intensity
8e+05
6e+05
4e+05
2e+05
0e+00
400
800
1200
1600
M/Z
B
Scatterplot of predicted elution time
Fitting R2: 0.852
Novel peptide residual Z score: 7.38
Number of peptides: 1048
Pred. hydrophobicity
60
40
20
0
0
25000
50000
75000
Experimental scan number
C
Distributions of residuals from best−fit line
of predicted RT vs Expt. scan number
Line: Z score of novel peptide
Z: 7.38
count
200
100
0
−10
−5
0
5
10
Z scores of residuals from best linear fit
